# Adaptive mechanism-based grey wolf optimizer for feature selection in high-dimensional classification

**DOI:** 10.1371/journal.pone.0318903

**Published:** 2025-05-16

**Authors:** Genliang Li, Yaxin Cui, Jingyu Su

**Affiliations:** 1 New Engineering Industry College, Putian University, Putian, Fujian, China; 2 Engineering Research Center of Big Data Application in Private Health Medicine, Fujian Province University, Putian, Fujian 351100, China; 3 Putian Science and Technology Plan Project (Putian Electronic Information Industry Research Institute), Putian, Fujian 351100, China; Ethics InfoTech, INDIA

## Abstract

Feature Selection (FS) is a crucial component of machine learning and data mining. Its goal is to eliminate redundant and irrelevant features from a datasets, thereby enhancing the classifier's performance. The Grey Wolf Optimizer (GWO) is a well-known meta-heuristic algorithm rooted in swarm intelligence. It is widely used in various optimization problems due to its fast convergence and minimal parameter requirements. However, in the context of solving high-dimensional classification problems, GWO’s global search capability is limited, and it is susceptible to getting trapped in local optima. To address this, we introduce an Adaptive Mechanism-based Grey Wolf Optimizer (AMGWO) for FS in high-dimensional classification. This approach encompasses a novel nonlinear parameter control strategy to balance exploration and exploitation effectively, thereby preventing the algorithm from converging prematurely. Additionally, an adaptive fitness distance balancing mechanism is proposed to prevent premature convergence and enhance search efficiency by selecting high-potential solutions. Lastly, an adaptive neighborhood mutation mechanism is designed to adjust mutation intensity adaptively during the search process, allowing AMGWO to more effectively find the global optimum. To validate the proposed AMGWO method, we assess its performance on 15 high-dimensional datasets and compare it with the original GWO and five of its variants in terms of classification accuracy, feature subset size, and execution speed, thus confirming the superiority of AMGWO.

## 1. Introduction

The process of Feature Selection (FS) is a critical component of data reprocessing, with the purpose of reducing data dimensionality and redundancy. This is achieved by extracting features that are meaningful for model prediction, ultimately leading to improvements in model performance and interpretability [1]. In the realm of machine learning and data mining, FS has found widespread application in solving practical problems, including extracting fetal electrocardiogram signals [2], gender detection from voice data [3], biological data analysis [4], and intelligent facial emotion recognition [5]. The implementation of effective FS significantly improves the learning efficiency and prediction accuracy of models.

The FS problem is known to be NP-hard, with the search space growing exponentially as the dimension increases, making exhaustive search impractical. To enhance the search efficiency of FS algorithms, numerous methods have been proposed by scholars. FS methods are typically categorized into three groups: filter methods, wrapper methods, and embedded methods [6]. Among these, the wrapper method is widely favored for its superior classification ability, and it is the focus of this work. This method primarily comprises three components: classifier, feature subset evaluation, and search technique [7]. Of these, an effective search technique is vital for the performance of FS algorithms. It is noteworthy that Metaheuristic methods (MH) such as Particle Swarm Optimization (PSO) [8], Differential Evolution (DE) [9], Genetic Algorithm (GA) [10], Artificial Bee Colony Algorithm (ABC) [11], Harris Hawk Optimization (HHO) [12], Whale Optimization Algorithm (WOA) [13], Moth-Flame Optimization (MFO) [14], Snake Optimizer (SO) [15], Binary Improved ChOA Algorithm (BICHOA) [16], Binary Improved White Shark Optimizer (BIWSO) [17], Opposition-based sine cosine optimizer (IBSCA) [18], Improved Binary DJaya Algorithm (IBJA) [19], among others, have been extensively utilized in the realm of FS. In addition, MH are also widely used in other fields, for example, Structural Optimization [20,21], Economic Load Dispatch Problem [22], Patient Admission Scheduling Problem [23], Intrusion Detection Systems [24], Point Cloud Registration [25], and others. Meanwhile, some literatures of great research value are as follows: Metalearning-Based Alternating Minimization Algorithm [26], HyGloadAttack [27], Novel method for reliability optimization [28], Topology optimization [29], Multi-objective robust optimization model [30], Computational intelligence-based classification system [31], Near miss prediction [32], SLNL [33], Multi-Character Classification [34], and TMFF [35].

The MH method offers four key advantages in addressing such issues [36]: simplicity, flexibility, absence of a derivation mechanism, and the capability to evade local optima. Typically, the MH algorithm's search process comprises two stages [37]: the initial stage involves exploration, while the subsequent stage involves exploitation. During the exploration phase, the algorithm thoroughly navigates the search space to uncover diverse solutions to the problem. In the exploitation phase, the algorithm leverages local information to generate improved solutions, typically in the proximity of the current solution. Excessive exploration slows down the algorithm's convergence, while excessive exploitation raises the risk of getting trapped in local optima. Striking a balance between exploration and exploitation is a primary objective in algorithm design to achieve optimal performance.

The Grey Wolf Optimizer (GWO) is a meta-heuristic optimization algorithm inspired by the collective hunting behavior of grey wolves [38]. By emulating the social hierarchy and hunting strategies of grey wolves, GWO can effectively balance global search and local exploitation. However, when confronted with high-dimensional and intricate optimization problems, the original GWO algorithm is susceptible to getting trapped in local optima and demonstrates certain limitations in terms of convergence speed and accuracy. As a result, various GWO variants have been proposed by researchers, and they have been successfully applied across a range of real-world fields, as detailed in Table 1.

**Table 1:** Details of multiple GWO algorithm variants.

Author	Variants	Improvement strategier	Field of application	Year
Emary et al. [39]	bGWO	1. Stochastic crossover strategy.	Feature selection	2016
		2. Sigmoidal function is used to squash the continuous updated position.		
Zhao et al. [40]	QCGWORS	1. Propose quantum computing strategy.	Feature selection and classification	2020
		2. Propose uncertain symmetry rough set		
		strategy.		
Zhang et al. [1]	BABCGWO	1. Present a novel ABC framework.	Feature selection	2021
Wang et al. [41]	ABGWO	1. Introducing a random individual and	Feature selection	2022
		Semi-random selection.		
		2. An adaptive coefficient is introduced.		
Paharia et al. [42]	MOCBGWO	1. Propose a discrete search space strategy.	Facial expression recognition and Feature selection	2022
Wang et al. [43]	BFLGWO	1. Combining the top wolves with foraging and following.	Feature selection	2023
		2. Combining the ordinary wolves with		
		Levy flight.		
		3. Novel V-shaped linear transfer functions.		
Li et al. [44]	IGWO-WFs	1. Propose multi-modal adaptive function, sigmoid function and autoregressive function strategies.	Unmanned Aerial Vehicles path planning	2023
He et al. [45]	LGWO-SVR	1. Levy flight strategy and support vector regression (SVR) are proposed.	Dam deformation	2023
Ou et al. [46]	pGWO-CSA	1. The nonlinear convergence factor is	Robot path planning	2023
		proposed.		
		2. Design new location update strategies.		
		3. Cloning and super-mutation strategies are introduced.		
Chang et al. [47]	MSGWO	1. Propose a random opposition-based	Fund performance and Feature selection	2024
		learning strategy.		
		2. A new nonlinear convergence factor is		
		proposed.		
		3. New update rules are proposed based on mutation operators.		
Premkumar et al. [48]	KCGWO	1. Proposed K-means clustering-based grey wolf optimizer.	Data mining and K-means clustering	2024
Zhao et al. [49]	GSGWO	1. Introduce the chaotic map.	Path Planning of Obstacle-Crossing Robot	2024
		2. The convergence strategy of the golden		
		sine optimizer is introduced.		
Lian et al. [50]	CGWO	1. Propose a probability-based mapping	Crowdsourcing applications and Mobile edge computing	2024
		scheme.		
		2. Introduce a new position update strategy.		
Zhang et al. [51]	AFGWO	1. Propose quadratic interpolation and elite reverse learning.	Engineering design problems	2024
		2. Introduce new position update strategies.		
Zhu et al. [52]	WNT-GWO	1. A dynamic weighted grey Wolf position updating rule is proposed.	Parameter identification of proton exchange membrane fuel cells	2024
Liang et al. [53]	ACGWO	1. Proposed nonlinear factor.	Daily streamflow prediction	2024
		2. Chaos condition and adaptive update mechanism are introduced.		

As per the No Free Lunch Theorem (NFL) [54], it is acknowledged that no single optimization algorithm excels across all problems. Hence, it is imperative to enhance and fine-tune algorithms to suit specific problems. This paper introduces a new variant of the GWO to improve its FS ability, addressing its current limitations. The key contributions of this paper are outlined below:

(1) A new nonlinear parameter control strategy is introduced to effectively balance algorithm exploration and exploitation.
(2) An adaptive fitness distance balance mechanism was proposed to accelerate the convergence speed of the algorithm.
(3) An adaptive neighborhood mutation mechanism is designed to fully consider the information exchange between α, β, δ wolves and the current global optimal solution. This allows the algorithm to explore the solution space more effectively and escape local optima.
(4) Fifteen data sets were used to test the performance of the AMGWO algorithm, and the effectiveness and robustness of the AMGWO algorithm were verified.

The paper is organized as follows: Section 2 outlines the FS problem and explains the fundamental principles of the original GWO. Section 3 provides a detailed introduction to the AMGWO algorithm. The experimental setup is described in Section 4. Section 5 presents comprehensive experiments and analysis to verify the effectiveness and performance advantages of the AMGWO algorithm in FS. Lastly, Section 6 concludes the manuscript and discusses future research directions and potential applications.

## 2. Preliminaries

In this section, we briefly review the feature selection problem and the original grey wolf optimizer.

### 2.1 Feature selection

Consider a dataset *S* containing *D* features and *L* samples. The FS problem aims to choose *d* features (where *d* < *D*) from the entire feature set in order to optimize the objective function Y(·). For practical reasons, the solution to the FS problem *X* is often represented as a binary string, with each bit indicating whether a feature is chosen for model construction.


$$X=\left(x_{1},x_{2},\ldots ,x_{D}\right),{\;x}_{i}\in 0,1$$ (1)

where, $x_{i}=1$ means that the *i*^th^ feature is selected in the subset X, otherwise it is not selected.

In FS problems, Y(·) often refers to the accuracy or error rate of a classification. If Y(·) represents the classification error rate, the FS problem can be expressed by the mathematical formula (2).


$$\begin{array}{l} minY(X)\\ s.t.X=(x_{1},x_{2},\ldots ,x_{D}) \end{array}$$ (2)

### 2.2 Gray Wolf Optimizer

**Fig 1:**
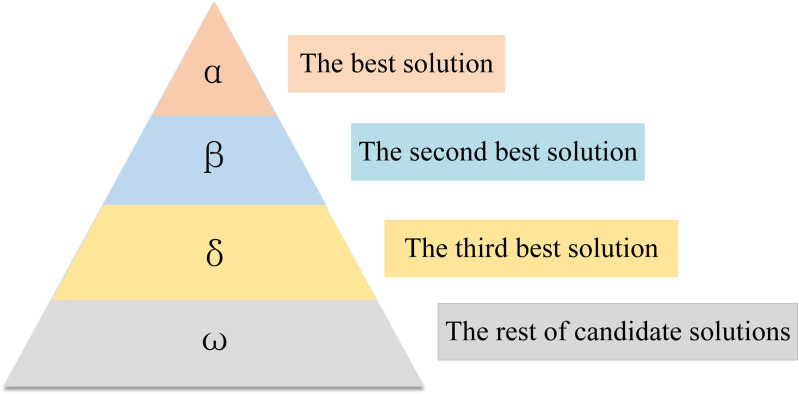
Grey Wolf hierarchy.

Proposed by Mirjalili et al. [38], the Grey Wolf Optimizer is based on the social hierarchy and hunting behavior of grey wolf populations. As illustrated in Fig 1, the grey wolf population adheres to a strict hierarchy consisting of four levels: α, β, δ, and ω. Among them, α wolves, as the supreme leaders, significantly influence crucial aspects such as hunting and habitat selection. β wolves assume a secondary role, working closely with α wolves, and providing crucial support in decision-making, planning group actions, and hunting strategies. δ wolves closely follow, guarding territorial boundaries and alerting the pack when threatened, while following the lead of both α and β wolves. Although ω wolves may seem to have a lower status, they play an essential role in maintaining the pack’s internal balance under the guidance of the α, β, and δ wolves.

In the GWO mathematical model, the optimal solution can be likened to the α wolf, the sub-optimal solution to the β wolf, the third optimal solution to the δ wolf, and the remaining candidate solutions to ω wolves. By utilizing the location information of α, β, and δ wolves as search guidance, we can direct the entire search process towards the optimal solution. This simulates a cooperation mechanism among them, leading to an effective solution for the complex problem.

The behavior of the grey Wolf while searching for prey is abstracted as mathematical formulas (3) and (4).


$$D=\left|C\times X_{p}(t)-X(t)\right|$$ (3)

$$X(t+1)=X_{p}(t)-A\times D$$ (4)

where, D represents the distance between the prey and the wolf during the search process, C and A represent the coefficient vectors, which are calculated by formula (5) and formula (6), respectively. $X_{p}(t)$ represents the current position of the prey, X(t) represents the position of the gray wolf in the current iteration, and X(t+1) represents the new position of the gray wolf in the next iteration.


$$A=2\times a\times r_{1}-a$$ (5)

$$C=2\times r_{2}$$ (6)

The a said coefficients of a linear decrease from 2 to 0, $r_{1}$ and $r_{2}$ random number from 0 to 1.

In summary, the mathematical model of the entire hunting behavior of the gray Wolf is as follows:


$$D_{\alpha }=\left|C_{\alpha }\times X_{\alpha }-X\right|,\;D_{\beta }=\left|C_{\beta }\times X_{\beta }-X\right|,\;D_{\delta }=\left|C_{\delta }\times X_{\delta }-X\right|$$ (7)

$$X_{1}=X_{\alpha }-A_{1}\times D_{\alpha },\;X_{2}=X_{\beta }-A_{1}\times D_{\beta },\;X_{3}=X_{\beta }-A_{1}\times D_{\beta }$$ (8)

$$X(t+1)={\frac{1}{3}\times (X}_{1}+X_{2}+X_{3})$$ (9)

**Fig 2:**
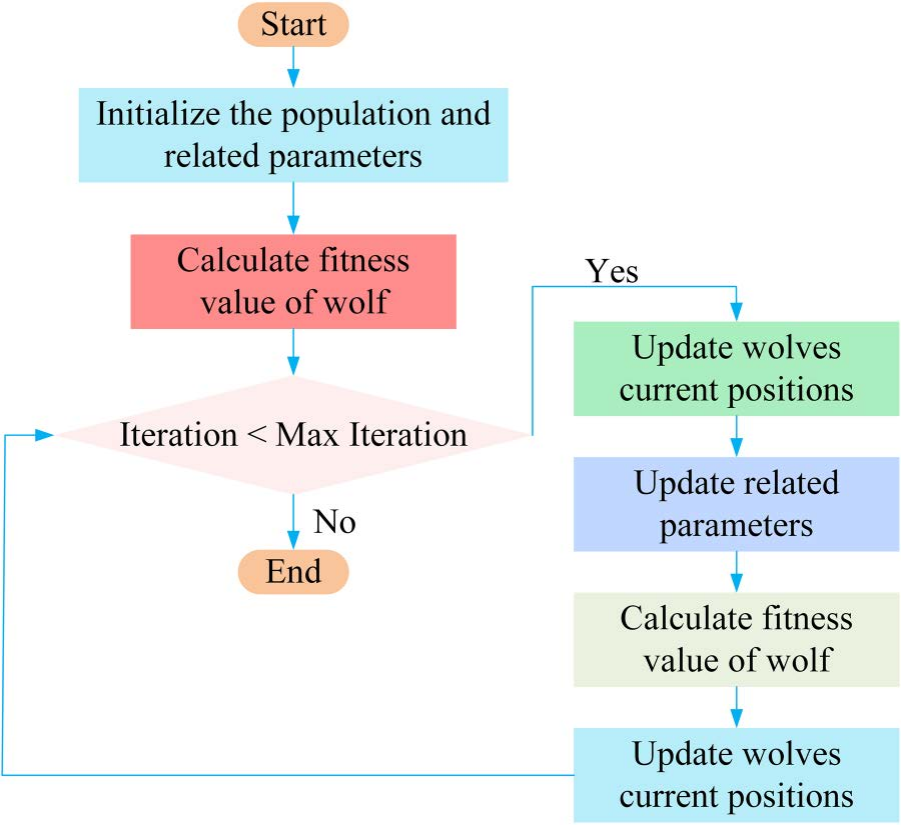
The flowchart of GWO.

It is worth emphasizing that when the parameter A>1, the grey wolf moves farther away from the prey, which helps the algorithm explore the search space more widely. On the other hand, if A<1, the grey wolf closely surrounds the prey, promoting rapid convergence to the global optimal solution. Clearly, the dynamic adjustment of the grey wolf's position is guided by three core levels: α, β, and δ. Fig 2 shows the flow diagram of the GWO.

## 3. The proposed AMGWO method

In this section, we introduce a novel Grey Wolf Optimizer with adaptive mechanisms, incorporating three primary adaptive mechanisms: the adaptive parameter control mechanism (APCGWO), the adaptive fitness distance balance mechanism (AFDBGWO), and the adaptive neighborhood mutation mechanism (ADVGWO).

### 3.1 Adaptive parameter control mechanism (APCGWO)

**Fig 3:**
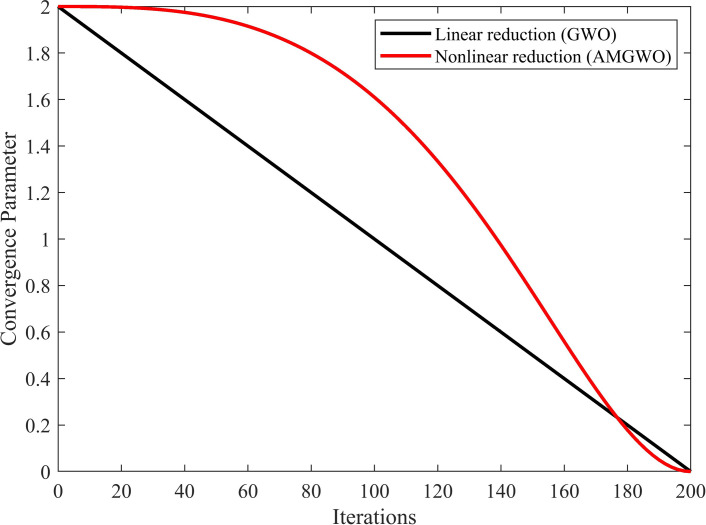
Comparison of GWO and AMGWO convergence factors.


The strategy of controlling the nonlinear parameter effectively balances the exploration and exploitation phases [55]. In the original GWO, the slow convergence rate or low convergence accuracy is partly due to the linear reduction of the convergence factor. To tackle this issue, this paper introduces a new nonlinear reduction strategy, presented in Eq. (10). As depicted in Fig 3, the convergence factor decreases nonlinearly from 2 to 0. The graph shows that at the start of the iteration, the curve is flat, indicating effective exploration of the entire search space by the AMGWO algorithm. In later iterations, the curve decays rapidly, signifying quick convergence of the algorithm.

$$a_{new}=2-2\times \sin\left(\frac{\pi }{2}\times {\left(\frac{t}{T}\right)}^{3}\right)$$ (10)

### 3.2 Adaptive fitness distance balancing mechanism (AFDBGWO)

The Fitness Distance Balancing (FDB) method focuses on balancing the fitness of a solution candidate with its distance from the current optimal solution in the search space [56]. This strategy aims to mitigate the early convergence issue of metaheuristic search algorithms and enhance search efficiency by selecting solution candidates with high potential. In this context, Fitness (F) typically denotes the quality of the solution; for minimization problems, a solution with higher fitness corresponds to a lower objective function value. The Distance metric (DP) captures the distance between the candidate and the best solution in the current population. This paper employs the Euclidean distance for calculation purposes:


$$DP=\sqrt{\sum_{i=1}^{n}\;(x_{i}-x_{best}{)}^{2}}$$ (11)

where, $x_{i}$ represents the position coordinates of the solution candidate, $x_{best}$ denotes the position coordinates of the optimal solution, and n is the number of populations.

The FDB method computes a score for each solution candidate, which combines both fitness and distance factors. The calculation formula of scoring $S_{FDB}$ can use the following ways:


$$S_{FDB}=normF+normDP$$ (12)

where, normF and normDP represent represent the normalized values of fitness and distance, respectively. The solution candidate with the highest FDB score is selected for the subsequent search operation during the selection process. This process allows the algorithm to maintain a certain degree of exploration through distance values while also leveraging known better solutions through fitness values. The FDB method effectively enhances the performance of metaheuristic algorithms by comprehensively considering the fitness and diversity of solution candidates, particularly in complex problems with multiple local optima. In this paper, we introduce the FDB mechanism to redesign the GWO algorithm and incorporate an adaptive strategy. The improved Grey Wolf step update method is as follows:


$$D_{\alpha }=\left|C_{\alpha }\times X_{\alpha }-X^{\prime }\right|,\;D_{\beta }=\left|C_{\beta }\times X_{\beta }-X^{\prime }\right|,\;D_{\delta }=\left|C_{\delta }\times X_{\delta }-X^{\prime }\right|$$ (13)

where, $X^{\prime }$ is the location of the improved gray wolves choose way, provide the formula (14) to choose, according to the number of iterations from the original method of adaptive or individual based on FDB score choice step updates, such not only can maintain the population diversity, can also speed up the convergence speed and accuracy of the algorithm.


$$\begin{array}{c} \left\{ \begin{array}{c} X^{\prime }=X,\;if\;mod(t,2)==0\\ X^{\prime }=X_{FDB},\;else \end{array}\right.\; \end{array}$$ (14)

Take this mod representative, t said the current number of iterations,$\;X_{FDB}$ FDB individuals with the highest scores.

### 3.3 Adaptive neighborhood mutation mechanism (ADVGWO)

In order to enhance the global search capabilities of the GWO algorithm and prevent the occurrence of local optima in later iterations, this study introduces an adaptive neighborhood mutation mechanism. This mechanism takes into account the information exchange between α, β, δ wolves and the current global optimal solution. Its goal is to dynamically adjust the mutation intensity during the search process, enabling the algorithm to explore the solution space more effectively and avoid local optima. Initially, a mutation intensity factor is defined to measure the disparity between different ranks of wolves and the global optimal solution, as shown in Eq. (15). Subsequently, utilizing the calculated mutation intensity, the mutation operation is performed in the neighborhood of each level of wolves using Eq. (16). This step is designed to dynamically modify the search direction and step size of the wolves based on their relative positions to the global optimal solution, thereby achieving adaptive domain variation. This mechanism not only strengthens the algorithm's exploration capability but also maintains the efficiency of utilizing known information, offering a novel approach for addressing intricate optimization problems.


$$r_{1}=X_{best}-X_{1},\;r_{2}=X_{best}-X_{2},\;r_{3}=X_{best}-X_{3}$$ (15)

$$X_{1}=X_{1}+r_{1}\times randn,\;X_{2}=X_{2}+r_{2}\times randn,\;X_{3}=X_{3}+r_{3}\times randn$$ (16)

where, randn denotes a standard normal random number. In summary, the pseudocode of AMGWO is shown in **Algorithm 1**.

Algorithm 1 AMGWO

1:    Begin

2:    Initialize the AMGWO parameters

3:    Calculate the fitness of each grey wolf

4:    Select α, β, and δ

5:    For t*** ***=*** ***1:Tmax

6:      The adaptive parameters are updated by Eq. (10)

7:      Step size updates by Eq. (13)

8:      Grey wolf adaptive domain variation by Eq. (16)

9:      For each grey wolf

10:       Change the positions by Eq. (9)

11:      End for

12:      Calculate the fitness of each grey wolf

13:      Update α, β, δ wolf

14       t* *=* *t* *+* *1

15:    End for

16:    Return best solution

17:    End

// Tmax denotes the maximum number of iterations

### 3.4 Time complexity analysis

For the same problem, we have a range of algorithmic solutions, each with its own set of advantages and disadvantages directly impacting execution efficiency and overall program performance [57]. The primary objective of algorithm analysis is to pinpoint the most suitable and optimized algorithm. In this study, we utilize the Big O notation as an assessment tool to gauge the time complexity of the algorithm [58,59], and perform a comparative analysis between AWGWO and GWO.

The specific application steps of the O-order method [60] are briefly described as follows:

Simplified constant term: First, all additive constants in the running time of the algorithm are uniformly simplified to a constant 1 for the convenience of subsequent analysis.

Keep the highest order terms: In the adjusted running time function, only the highest order terms that have the greatest impact on the complexity are kept, and other lower order terms are ignored.

Non-essential constants are removed: If the highest order term exists and its coefficient is not 1, the coefficient is further removed and only its order is retained as the O order representation.

Following the above steps, the time complexity analysis result of GWO is O(n× d× T), where n, d, and T represent the population size, dimension, and iteration number, respectively. It is worth noting that in the calculation process of AMGWO, no new loop structure is introduced, and no fundamental changes are made to the original loop order. Therefore, its time complexity analysis result is consistent with that of GWO, which is also O(n× d× T).

## 4. Experimental setup

In this section, we first introduce the transformation function. Second, we elaborate on the fitness function design. Next, we introduce the datasets and evaluation metrics selected in this paper. Finally, we report the chosen contender parameter Settings.

### 4.1 Transformation functions

The traditional GWO excels at solving continuous search space problems. However, it encounters challenges when applied to problems such as FS, which involve binary optimization with solutions restricted to binary values {0,1}. To address this issue, researchers have introduced the concept of a transformation function, aimed at adapting the original capabilities of the Grey Wolf algorithm in the continuous search space to the binary domain. Among various transformation functions, the sigmoid function σ(⋅) stands out due to its unique properties. This function converts continuous input values into discrete values by mapping them to the range (0, 1) and then thresholding them to binary values (typically using 0.5 as the threshold). It is important to note that this approach has been proven effective in numerous GWO variants [41,42]. In our study, we similarly employed the sigmoid function as the transformation tool. The transformation function is defined as follows:


$$x_{i,j}\;=\left\{\begin{array}{l} 1,\sigma (x_{i,j})>r\\ 0,otherwise \end{array}\right.$$ (10)

where, $x_{i,j}$ represents the continuous value corresponding to the *i*^th^ search agent in dimension d. r is a random number between 0 and 1. The function *σ*(⋅) is regarded as a concrete example of the s-type function (Sigmoid function) [61]. The mathematical expression of the function is as follows:


$$\sigma (\alpha )=\frac{1}{1+e^{-10(\alpha -0.5)}}$$ (11)

### 4.2 Fitness function

In our experiments, we utilized the wrapper technique to execute each FS algorithm. The wrapper approach offers the distinct advantage of using the classifier's performance as the criterion for selecting features, resulting in an efficient and accurate FS process. To enhance the quality of the selected features, we specifically opted for the wrapper method based on the KNN [62] classifier, with the K value set to 5 (K = 5) to achieve superior classification results. To mitigate the risk of overfitting, we recommend employing a 10-fold cross-validation technique when working with real data sets [63]. In wrapper FS methods, the fitness function directly mirrors classification performance. The specific calculation formula for the fitness function used in this paper is elaborated in Eq. (20).


$$fitness=\frac{\sum_{i=1}^{10}\;error_{i}}{10}$$ (12)

where, $error_{i}$ represents the classification error rate when *i*^th^ runs. The mathematical model is as follows:


$$error_{i}=\frac{The\;number\;of\;misclassified\;samples}{Total\;number\;of\;samples}$$ (13)

### 4.3 Datasets

In order to evaluate the performance of the proposed FS algorithm, we conducted experiments using 15 benchmark datasets. Table 2 provides an overview of the key parameters of these datasets, including the number of features, samples, and categories. These datasets are characterized by high dimensionality and small sample size, which are commonly encountered in FS literature. They have been sourced from Arizona State University and Jilin University and encompass a wide range of data types, such as microarray gene expression, image (face) detection, and email text. It’s worth noting that the datasets have been pre-processed by their respective providers.

**Table 2:** The dataset used in the experiments.

Dataset	Features	Samples	Classes
Yale	1024	165	15
ORL	1024	400	40
COIL20	1024	1440	20
Colon	2000	62	2
SRBCT	2308	83	4
warpAR10P	2400	130	10
warpPIE10P	2420	210	10
DBWorld	4702	64	2
Leukemial	5327	72	3
DLBCL	5469	77	2
ALLAML	7129	72	2
Pixraw10P	10000	100	10
orlraws10P	10304	100	10
Prostate	10509	102	2
Leukemia2	11225	72	3

### 4.4 Evaluation metrics

In order to ensure the reliability of the FS results, we performed 10 independent runs to evaluate the FS method. Our focus was on assessing classification accuracy and feature subset dimension. It’s important to note that in our experiments, each FS method produced a distinct feature subset for each dataset in every run. To validate the effectiveness of our proposed method, we employed a range of quantitative indicators for evaluation.

(1) Best: minimum classification error/feature subset size of all solutions obtained by running the algorithm 10 times.
(2) Worst: maximum classification error/feature subset size of all solutions obtained by running the algorithm 10 times.
(3) Mean [64]: the average classification error rate/the size of the feature subset of all solutions obtained by running the algorithm 10 times.
(4) Standard deviation (Std): The standard deviation is calculated on the set of all final solutions obtained by running the algorithm 10 times. It is an important indicator to measure the stability and robustness of the optimizer, and the mathematical expression is as follows:

$$Std=\sqrt{\frac{1}{H-1}\sum (best_{h}-Mean{)}^{2}}$$ (14)

where, H is the number of times the algorithm is run for the FS problem. $best_{h}$ is the best solution to run h times.

### 4.5 Competitor parameter settings

In order to evaluate the performance of the proposed AMGWO algorithm, we conducted a comparative analysis with six other optimization algorithms, including the original GWO and its six variants: GWO [38], GNHGWO [65], BABCGWO [1], SOGWO [66], EGWO [67], and AGWO [68]. The parameter settings of these optimization algorithms are detailed in Table 3. The maximum number of iterations and the population size were standardized at 100 and 30, respectively. Each algorithm was independently executed 30 times to ensure a fair comparison. Subsequently, the standard deviation (Std), best, worst, and Mean values were calculated and recorded, with the best result being highlighted in bold.

**Table 3:** Parameter Settings for competitive algorithms.

Algorithms	Name of the parameter	Value of the parameter
GWO	a	[0,2]
GNHGWO	a	[0,2]
BABCGWO	a	[0,2]
SOGWO	a	[0,2]
EGWO	a	(0, 1)
AGWO	None	None
AMGWO	a	[0,2]

## 5. Results and discussion

### 5.1 Comparison with competitor algorithms

In this section, the proposed AMGWO is compared with six other competitive algorithms in terms of classification error rate, feature subset size, and running time. This comparison aims to verify the effectiveness and efficiency of AMGWO in the context of FS for classification tasks.

#### 5.1.1 Accuracy analysis.

**Fig 4:**
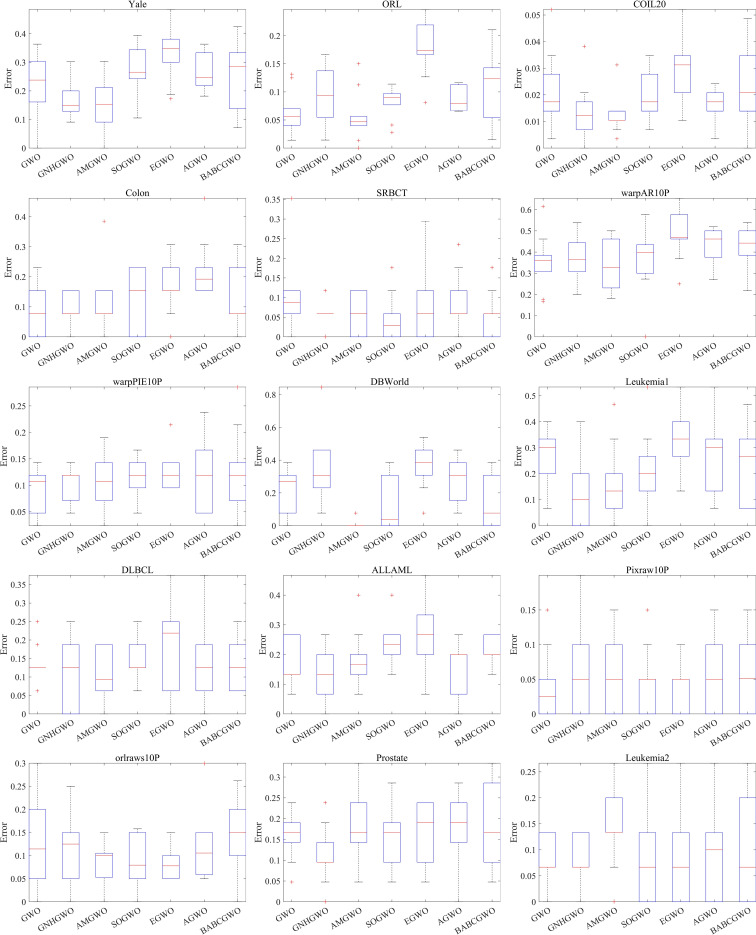
Boxplots of classification error rates for all algorithms.

The classification error rates of AMGWO and six other competing algorithms are presented in Table 4. The superior results are highlighted in bold. Across most datasets, AMGWO outperforms the other algorithms in terms of classification error rates. The smaller standard deviation of the classification error rate indicates that the algorithm is more stable and robust. Furthermore, the complexity of the problem is directly linked to its dimensionality. As the problem dimensionality increases, AMGWO exhibits the statistically smallest error rate, suggesting that its classification performance is least affected by higher dimensionality. To further illustrate the effectiveness of AMGWO in terms of classification error rate, we have included boxplots of all algorithms in Fig 4. It is evident from the boxplot positions that AMGWO outperforms the other algorithms in terms of stability and robustness.

**Table 4:** Comparison of classification error rates for different competitor algorithms, with the top-ranked results shown in bold.

Datasets	Metrics	GWO	GNHGWO	BABCGWO	SOGWO	EGWO	AGWO	AMGWO
Yale	Best	**0.0000E + 00**	9.0909E-02	7.1429E-02	1.0526E-01	1.7241E-01	1.8182E-01	**0.0000E + 00**
	Worst	3.6364E-01	**3.0303E-01**	4.2424E-01	3.9394E-01	4.8485E-01	3.6364E-01	**3.0303E-01**
	Mean	2.2055E-01	1.7501E-01	2.6004E-01	2.6965E-01	3.3816E-01	2.6809E-01	**1.4592E-01**
	Std	1.1442E-01	7.3245E-02	1.2085E-01	8.7935E-02	1.0525E-01	**6.4283E-02**	9.9995E-02
ORL	Best	1.4085E-02	1.4286E-02	1.5152E-02	2.7778E-02	8.1081E-02	6.5789E-02	**0.0000E + 00**
	Worst	1.3158E-01	1.6667E-01	2.1053E-01	**1.1392E-01**	2.4675E-01	1.1667E-01	1.5000E-01
	Mean	6.2094E-02	9.6212E-02	1.1127E-01	8.2722E-02	1.7714E-01	8.7906E-02	**5.5952E-02**
	Std	4.0035E-02	5.2474E-02	6.6776E-02	2.8449E-02	4.9178E-02	**2.1504E-02**	4.4322E-02
COIL20	Best	3.4722E-03	**0.0000E + 00**	**0.0000E + 00**	6.9444E-03	1.0417E-02	3.4722E-03	3.4722E-03
	Worst	5.2083E-02	3.8194E-02	4.8611E-02	3.4722E-02	5.2083E-02	**2.4306E-02**	3.1250E-02
	Mean	2.1875E-02	1.3889E-02	2.4653E-02	1.9792E-02	2.9861E-02	1.6319E-02	**1.2500E-02**
	Std	1.4180E-02	1.0352E-02	1.4953E-02	9.2665E-03	1.2271E-02	**6.9541E-03**	7.3566E-03
Colon	Best	**0.0000E + 00**	**0.0000E + 00**	**0.0000E + 00**	**0.0000E + 00**	**0.0000E + 00**	1.5385E-01	**0.0000E + 00**
	Worst	2.3077E-01	**1.5385E-01**	3.0769E-01	2.3077E-01	3.0769E-01	4.6154E-01	3.8462E-01
	Mean	9.2308E-02	**9.2308E-02**	1.3077E-01	1.1538E-01	1.6923E-01	2.2308E-01	1.0769E-01
	Std	8.7330E-02	**4.8650E-02**	9.6282E-02	1.0415E-01	8.7330E-02	9.8976E-02	1.0999E-01
SRBCT	Best	**0.0000E + 00**	**0.0000E + 00**	**0.0000E + 00**	**0.0000E + 00**	**0.0000E + 00**	**0.0000E + 00**	**0.0000E + 00**
	Worst	3.5294E-01	**1.1765E-01**	1.7647E-01	1.7647E-01	2.9412E-01	2.3529E-01	**1.1765E-01**
	Mean	1.0000E-01	5.8824E-02	5.2941E-02	**4.7059E-02**	8.2353E-02	8.2353E-02	5.8824E-02
	Std	1.0017E-01	**3.9216E-02**	5.8496E-02	6.0753E-02	9.2801E-02	7.4407E-02	4.8029E-02
warpAR10P	Best	1.6667E-01	2.0000E-01	2.1739E-01	**0.0000E + 00**	2.5000E-01	2.6923E-01	1.8182E-01
	Worst	6.1538E-01	5.3846E-01	5.3846E-01	5.7692E-01	6.5385E-01	5.2000E-01	**5.0000E-01**
	Mean	3.5258E-01	3.7416E-01	4.2391E-01	3.6474E-01	4.7844E-01	4.3532E-01	**3.3357E-01**
	Std	1.3044E-01	1.0104E-01	9.6090E-02	1.5636E-01	1.1343E-01	**8.8293E-02**	1.1902E-01
warpPIE10P	Best	**2.3810E-02**	4.7619E-02	**2.3810E-02**	4.7619E-02	9.5238E-02	4.7619E-02	**2.3810E-02**
	Worst	**1.4286E-01**	**1.4286E-01**	2.8571E-01	1.6667E-01	2.1429E-01	2.3810E-01	1.9048E-01
	Mean	**9.2857E-02**	1.0238E-01	1.2473E-01	1.1463E-01	1.2857E-01	1.2619E-01	1.0714E-01
	Std	4.2666E-02	**2.9802E-02**	7.7591E-02	3.5467E-02	3.5846E-02	6.9234E-02	4.9245E-02
DBWorld	Best	**0.0000E + 00**	7.6923E-02	**0.0000E + 00**	**0.0000E + 00**	7.6923E-02	7.6923E-02	**0.0000E + 00**
	Worst	3.8462E-01	8.4615E-01	3.8462E-01	3.8462E-01	5.3846E-01	4.6154E-01	**7.6923E-02**
	Mean	2.0000E-01	3.4615E-01	1.4615E-01	1.3077E-01	3.6923E-01	2.6154E-01	**1.5385E-02**
	Std	1.4595E-01	2.1529E-01	1.5576E-01	1.6637E-01	1.4414E-01	1.3665E-01	**3.2434E-02**
Leukemial	Best	6.6667E-02	**0.0000E + 00**	6.6667E-02	**0.0000E + 00**	1.3333E-01	6.6667E-02	**0.0000E + 00**
	Worst	**4.0000E-01**	**4.0000E-01**	4.6667E-01	5.3333E-01	5.3333E-01	5.3333E-01	4.6667E-01
	Mean	2.6000E-01	**1.3333E-01**	2.4667E-01	2.1333E-01	3.2000E-01	2.6667E-01	1.5333E-01
	Std	**1.0634E-01**	1.4055E-01	1.5088E-01	1.4673E-01	1.2881E-01	1.3699E-01	1.4757E-01
DLBCL	Best	6.2500E-02	**0.0000E + 00**	6.2500E-02	6.2500E-02	**0.0000E + 00**	**0.0000E + 00**	**0.0000E + 00**
	Worst	2.5000E-01	2.5000E-01	2.5000E-01	2.5000E-01	3.7500E-01	3.7500E-01	**1.8750E-01**
	Mean	1.3125E-01	**1.0625E-01**	1.4375E-01	1.5625E-01	1.8125E-01	1.3750E-01	**1.0625E-01**
	Std	**5.4725E-02**	8.8634E-02	7.2469E-02	6.0739E-02	1.1581E-01	1.0121E-01	7.8229E-02
ALLAML	Best	6.6667E-02	**0.0000E + 00**	1.3333E-01	1.3333E-01	6.6667E-02	**0.0000E + 00**	6.6667E-02
	Worst	**2.6667E-01**	**2.6667E-01**	**2.6667E-01**	4.0000E-01	4.6667E-01	**2.6667E-01**	4.0000E-01
	Mean	1.7333E-01	**1.4000E-01**	2.1333E-01	2.3333E-01	2.7333E-01	1.6000E-01	1.8000E-01
	Std	7.1665E-02	7.9815E-02	**5.2587E-02**	7.8567E-02	1.3499E-01	8.9993E-02	9.9629E-02
Pixraw10P	Best	**0.0000E + 00**	**0.0000E + 00**	**0.0000E + 00**	**0.0000E + 00**	**0.0000E + 00**	**0.0000E + 00**	**0.0000E + 00**
	Worst	1.5000E-01	2.0000E-01	1.5000E-01	1.5000E-01	**1.0000E-01**	1.5000E-01	1.5000E-01
	Mean	**4.0000E-02**	6.0000E-02	5.5263E-02	4.5000E-02	4.0000E-02	6.0000E-02	5.5000E-02
	Std	5.1640E-02	6.9921E-02	5.5005E-02	4.9721E-02	3.1623E-02	5.6765E-02	**2.9721E-02**
orlraws10P	Best	**0.0000E + 00**	**0.0000E + 00**	**0.0000E + 00**	**0.0000E + 00**	5.0000E-02	5.0000E-02	**0.0000E + 00**
	Worst	3.0000E-01	2.5000E-01	2.6316E-01	1.5789E-01	**1.5000E-01**	3.0000E-01	**1.5000E-01**
	Mean	1.2288E-01	1.0579E-01	1.4220E-01	8.8017E-02	**8.6111E-02**	1.2199E-01	9.0789E-02
	Std	9.4400E-02	8.0289E-02	7.8535E-02	5.4200E-02	**4.0169E-02**	7.4148E-02	4.5854E-02
Prostate	Best	4.7619E-02	**0.0000E + 00**	4.7619E-02	4.7619E-02	4.7619E-02	**0.0000E + 00**	4.7619E-02
	Worst	**2.3810E-01**	**2.3810E-01**	3.3333E-01	2.8571E-01	**2.3810E-01**	2.8571E-01	3.3333E-01
	Mean	1.5714E-01	**1.1429E-01**	1.8095E-01	1.5238E-01	1.6190E-01	1.7143E-01	1.7619E-01
	Std	**5.5214E-02**	6.8088E-02	9.4708E-02	7.3771E-02	7.1693E-02	9.3098E-02	8.1092E-02
Leukemia2	Best	**0.0000E + 00**	**0.0000E + 00**	**0.0000E + 00**	**0.0000E + 00**	**0.0000E + 00**	**0.0000E + 00**	**0.0000E + 00**
	Worst	**1.3333E-01**	**1.3333E-01**	2.6667E-01	2.6667E-01	2.6667E-01	2.6667E-01	2.6667E-01
	Mean	**7.3333E-02**	**7.3333E-02**	8.6667E-02	9.3333E-02	9.3333E-02	9.3333E-02	1.4000E-01
	Std	4.9191E-02	4.9191E-02	9.9629E-02	8.9993E-02	8.9993E-02	9.5323E-02	**3.3367E-02**

#### 5.1.2 Feature number analysis.

**Fig 5:**
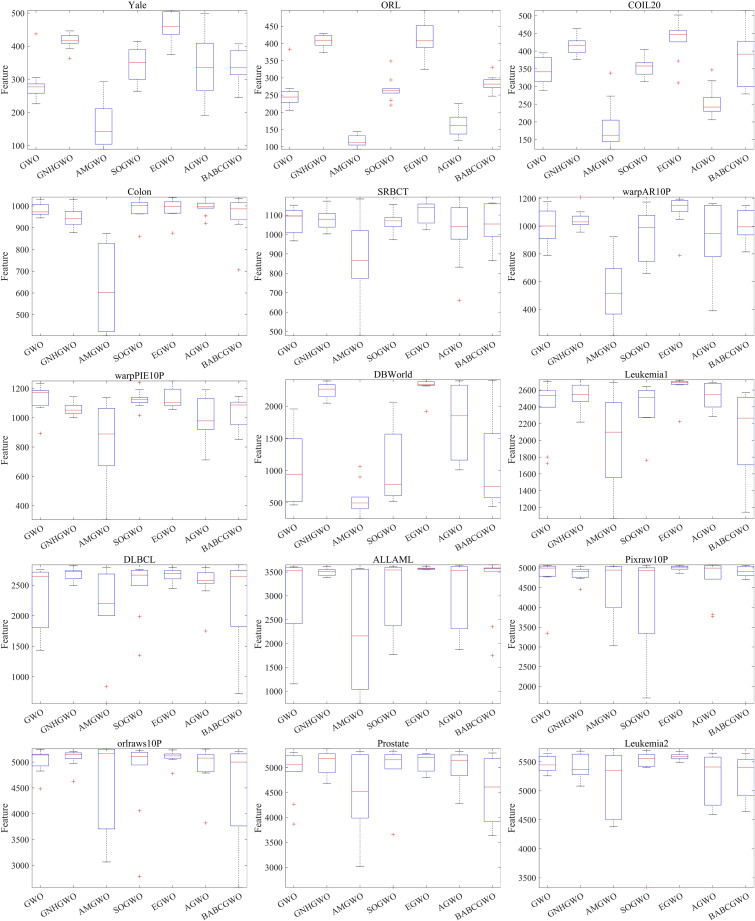
Boxplots of feature subset size for all algorithm.

The results for the feature subset sizes of AMGWO and six other competing algorithms are detailed in Table 5. The best results are indicated in bold. It is evident that in terms of feature subset size, AMGWO achieves the smallest feature subset across 11 datasets. On the 13 datasets, the average feature subset size is optimal. Furthermore, the standard deviation for most datasets with varying dimensions is minimal. Fig 5 displays a boxplot based on the feature subset size results. It is clearly evident from the boxplot that the position of AMGWO is lower than that of the other algorithms, indicating its effectiveness in reducing the feature subset size.

**Table 5:** Comparison of feature subset sizes for different competitor algorithms, and the top-ranked results are shown in bold.

Datasets	Metrics	GWO	GNHGWO	BABCGWO	SOGWO	EGWO	AGWO	AMGWO
Yale	Best	226.00	363.00	245.00	264.00	374.00	191.00	**89.00**
	Worst	437.00	446.00	407.00	414.00	508.00	499.00	**294.00**
	Mean	284.60	414.40	337.20	341.80	458.50	337.50	**158.40**
	Std	58.98	**23.15**	53.23	51.93	41.45	100.09	67.61
ORL	Best	206.00	373.00	247.00	221.00	325.00	119.00	**93.00**
	Worst	383.00	429.00	331.00	349.00	496.00	225.00	**144.00**
	Mean	255.60	407.30	284.30	267.90	417.20	167.20	**117.00**
	Std	48.84	18.57	22.06	34.58	52.51	36.01	**16.81**
COIL20	Best	289.00	377.00	279.00	314.00	310.00	206.00	**123.00**
	Worst	395.00	463.00	515.00	405.00	502.00	347.00	**338.00**
	Mean	344.90	416.80	378.40	356.00	431.80	256.30	**186.90**
	Std	37.84	**25.29**	76.65	25.57	53.71	44.65	68.47
Colon	Best	945.00	877.00	706.00	859.00	875.00	920.00	**403.00**
	Worst	1031.00	1030.00	1035.00	1041.00	1038.00	1041.00	**873.00**
	Mean	984.00	945.40	958.50	987.60	988.20	994.70	**617.50**
	Std	**28.46**	44.90	96.82	51.00	46.22	36.44	177.92
SRBCT	Best	967.00	1003.00	865.00	973.00	1024.00	660.00	**478.00**
	Worst	**1151.00**	1172.00	1162.00	1154.00	1193.00	1193.00	1183.00
	Mean	1072.20	1079.90	1044.70	1066.40	1118.20	1016.50	**866.70**
	Std	67.19	**56.71**	114.71	60.64	58.07	167.11	236.33
warpAR10P	Best	790.00	957.00	814.00	659.00	789.00	389.00	**209.00**
	Worst	1176.00	1210.00	1150.00	1174.00	1195.00	1165.00	**923.00**
	Mean	1006.30	1050.70	1003.40	943.70	1109.60	911.50	**541.70**
	Std	125.60	**68.78**	116.21	179.02	120.57	238.50	225.33
warpPIE10P	Best	892.00	1000.00	853.00	1016.00	1056.00	712.00	**304.00**
	Worst	1235.00	1145.00	1146.00	1239.00	1251.00	1191.00	**1139.00**
	Mean	1132.70	1061.20	1044.70	1126.80	1139.70	993.70	**853.70**
	Std	98.67	**44.66**	102.78	59.73	71.81	148.27	263.56
DBWorld	Best	467.00	2047.00	441.00	525.00	1919.00	1011.00	**256.00**
	Worst	1956.00	2390.00	2396.00	2061.00	2409.00	2391.00	**1065.00**
	Mean	1068.50	2241.10	1038.50	1012.90	2310.20	1748.30	**550.00**
	Std	523.58	**120.98**	651.26	543.52	141.20	592.60	249.97
Leukemial	Best	1726.00	2218.00	1143.00	1763.00	2225.00	2287.00	**1067.00**
	Worst	2705.00	2723.00	**2569.00**	2641.00	2716.00	2687.00	2688.00
	Mean	2387.50	2535.10	2081.20	2416.70	2644.50	2526.20	**1986.30**
	Std	341.23	150.36	512.83	266.98	**148.41**	158.65	595.86
DLBCL	Best	1435.00	2494.00	721.00	1350.00	2449.00	1750.00	**560.00**
	Worst	**2755.00**	2819.00	2834.00	2760.00	2793.00	2793.00	2791.00
	Mean	2314.60	2687.80	2264.30	2473.10	2666.40	2533.40	**2032.80**
	Std	516.60	**94.34**	684.04	458.29	107.96	301.87	760.46
ALLAML	Best	1154.00	3381.00	1747.00	1770.00	3546.00	1871.00	**740.00**
	Worst	3607.00	3611.00	3651.00	3616.00	3624.00	3646.00	**3579.00**
	Mean	2991.50	3493.40	3272.20	3004.60	3571.90	3121.40	**2267.80**
	Std	972.29	78.76	660.82	759.99	**24.46**	748.01	1218.09
Pixraw10P	Best	3349.00	4458.00	**1567.00**	1710.00	4864.00	3767.00	3030.00
	Worst	5055.00	**5032.00**	5068.00	5067.00	5062.00	5078.00	5042.00
	Mean	4800.90	4863.60	4589.70	**4068.00**	5001.60	4747.70	4541.50
	Std	520.59	171.26	1068.51	1282.34	**63.09**	512.68	755.44
orlraws10P	Best	4481.00	4626.00	**2572.00**	2785.00	4777.00	3822.00	3066.00
	Worst	5242.00	**5201.00**	5205.00	5229.00	5235.00	5252.00	5259.00
	Mean	5032.90	5077.50	**4431.70**	4776.00	5094.40	4916.00	4522.40
	Std	229.89	172.79	1070.46	780.95	**122.82**	416.49	918.78
Prostate	Best	3867.00	4687.00	3635.00	**2595.00**	4798.00	4280.00	3019.00
	Worst	5308.00	5380.00	5294.00	5330.00	**5285.00**	5321.00	5323.00
	Mean	4925.40	5096.40	4504.20	4769.50	5126.60	5009.90	**4431.10**
	Std	478.19	236.32	628.87	909.06	**187.78**	338.95	898.81
Leukemia2	Best	5258.00	5081.00	4642.00	**3331.00**	5489.00	4594.00	4379.00
	Worst	5643.00	5684.00	**5642.00**	5698.00	5676.00	5649.00	5726.00
	Mean	5458.10	5414.00	5278.20	5343.10	5587.50	5232.70	**5166.40**
	Std	147.56	202.25	356.62	713.41	**59.02**	434.11	545.57

#### 5.1.3 Convergence curve analysis.

**Fig 6:**
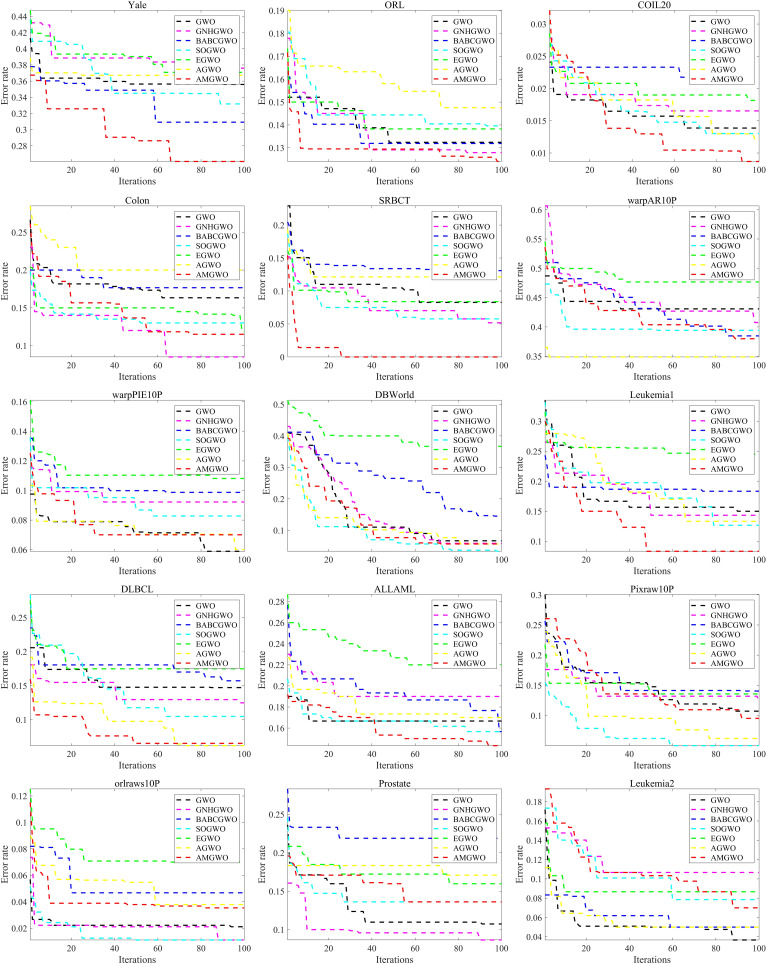
Convergence curves for all algorithms.

Please take note of the following text: The convergence curves of the seven algorithms on the 15 datasets are displayed in Fig 6. Each curve represents the average outcome of 10 runs for every iteration. These curves illustrate that AMGWO exhibits faster convergence and delivers higher-quality solutions compared to its counterparts across most datasets. While AMGWO may yield lower quality results than certain competing algorithms on the Prostate, Colon, and ALLAML datasets, it demonstrates superior convergence speed on these datasets. Overall, AMGWO outperforms the other six competing algorithms in terms of both convergence speed and solution quality.

#### 5.1.4 Runtime analysis.

**Fig 7:**
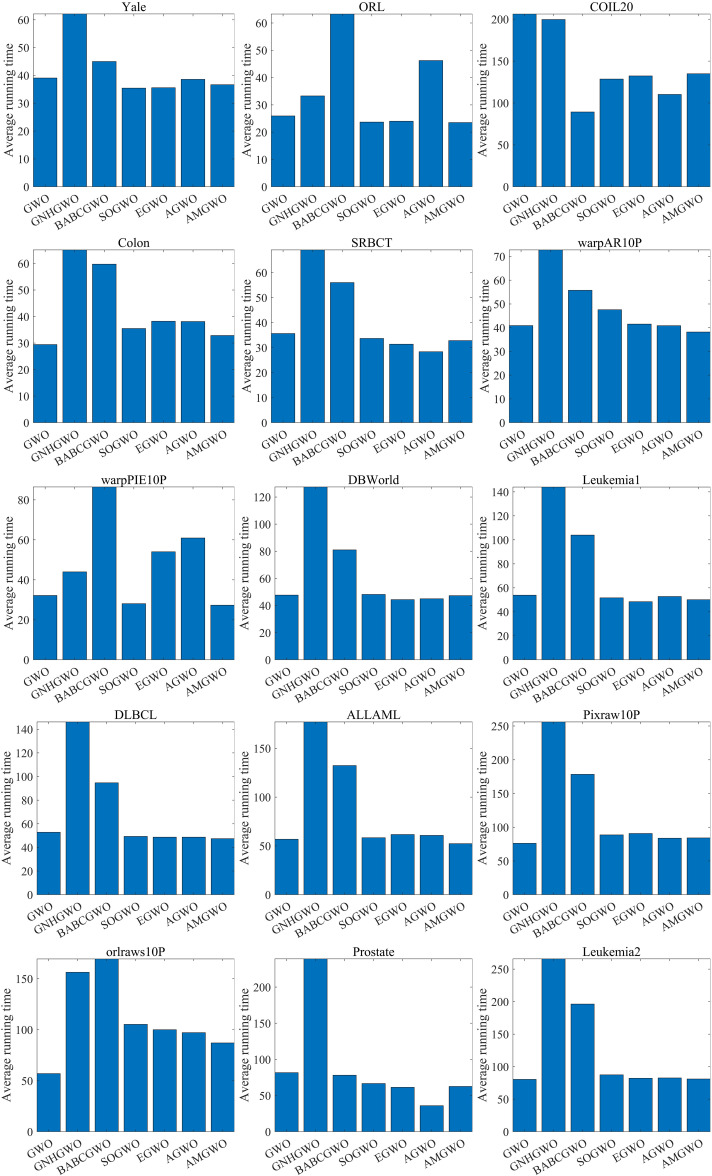
The average running time of all competing algorithms.

When dealing with high-dimensional datasets, the computational cost of FS algorithms mainly focuses on the evaluation of individual feature subsets. As shown in Fig 7, the running time of the AMGWO algorithm is lower than that of other competitors on most datasets, benefiting from the fact that each feature subset is evaluated only once per iteration in the AMGWO algorithm. Therefore, in terms of computation time, AMGWO outperforms the other algorithms.

### 5.2 Statistical tests

To further verify the excellent performance of AMGWO, we performed the Wilson rank sum test and the Friedman test on the classification error rate and feature subset size results obtained by the competitor algorithms.

#### 5.2.1 Wilcoxon rank sum test.

We utilized the Wilcoxon rank sum test [69] to compare the classification error rate and feature subset size results achieved by AMGWO and other competing methods, as presented in Table 6 and Table 7, respectively. A P-value lower than 0.05 indicates a significant difference between AMGWO and the other algorithms. Conversely, if the P-value is higher, there is no significant difference, and these non-significant results are highlighted in bold. It is evident from the tables that AMGWO exhibits notable distinctions from the competing algorithms across most datasets. Based on the analysis of the preceding experiments, it is evident that AMGWO significantly outperforms its competitors in terms of overall performance.

**Table 6:** The Wilcoxon rank sum test for classification error rates, where no difference is shown in bold.

Datasets	GWO	GNHGWO	BABCGWO	SOGWO	EGWO	AGWO	AMGWO
Yale	0.017281	0.005697	0.044912	0.019020	0.002807	0.009996	0.017281
ORL	0.049597	0.041174	**0.064022**	0.011173	0.000437	0.013983	0.049597
COIL20	0.006941	**0.078666**	0.015937	0.005005	0.002546	**0.116309**	0.006941
Colon	**0.084132**	0.009001	**0.516557**	0.007258	**0.073137**	0.005619	**0.084132**
SRBCT	0.035969	**1.000000**	0.006882	0.004951	**0.842362**	**0.630145**	0.035969
warpAR10P	**1.000000**	**0.426135**	**0.109433**	0.004044	0.022107	0.046393	**1.000000**
warpPIE10P	**0.051364**	**0.756435**	0.008486	**0.070103**	0.003534	0.006479	**0.051364**
DBWorld	0.003825	0.000146	0.017271	**0.099930**	0.000145	0.000189	0.003825
Leukemia1	0.006556	0.007286	0.018004	0.023434	0.019511	0.007681	0.006556
DLBCL	0.045978	0.009690	**0.329332**	0.019788	**0.104947**	**0.066815**	0.045978
ALLAML	**1.000000**	0.048265	0.019518	0.012884	0.011363	0.009378	**1.000000**
Pixraw10P	0.046776	0.009369	0.009374	**0.063087**	0.005652	0.009366	0.046776
orlraws10P	0.035887	**0.067195**	0.010563	0.009387	0.006403	0.041683	0.035887
Prostate	0.006971	0.009016	**1.000000**	**0.058801**	**0.846947**	0.009387	0.006971
Leukemia2	0.029031	0.029031	0.001756	0.018481	0.018481	0.023645	0.029031

**Table 7:** Wilcoxon rank sum test for feature subset sizes, where no difference is shown in bold.

Datasets	GWO	GNHGWO	BABCGWO	SOGWO	EGWO	AGWO	AMGWO
Yale	0.001706	0.000183	0.000330	0.000330	0.000182	0.001315	0.001706
ORL	0.000182	0.000181	0.000181	0.000181	0.000181	0.001480	0.000182
COIL20	0.000769	0.000183	0.000583	0.000440	0.000245	0.014019	0.000769
Colon	0.000181	0.000183	0.000440	0.000245	0.000183	0.000182	0.000181
SRBCT	**0.053903**	0.037635	**0.121225**	0.037635	0.011330	**0.212294**	**0.053903**
warpAR10P	0.000583	0.000183	0.000437	0.001706	0.000328	0.005777	0.000583
warpPIE10P	0.002202	**0.088973**	**0.064022**	0.008127	0.005795	0.027304	0.002202
DBWorld	0.014019	0.000183	0.021134	0.011330	0.000183	0.000246	0.014019
Leukemia1	0.014047	0.025748	**0.909722**	**0.104110**	0.002194	0.045155	0.014047
DLBCL	**0.677585**	0.017090	0.005708	0.014047	0.021134	**0.103980**	**0.677585**
ALLAML	0.016197	0.021229	0.025526	0.013043	0.017216	0.012122	0.016197
Pixraw10P	**0.384673**	**0.596563**	**0.939720**	**0.677585**	**0.088973**	0.042736	**0.384673**
orlraws10P	**0.909722**	**0.791337**	0.030731	**0.677585**	**0.850051**	**0.909722**	**0.909722**
Prostate	0.042718	0.014047	**0.677585**	**0.520523**	0.016197	0.027304	0.042718
Leukemia2	0.047268	**0.057075**	**1.000000**	**0.052052**	0.018571	0.008500	0.047268

#### 5.2.1 Friedman rank test.

We used the Friedman rank test [70] to compare the classification error rate and feature subset size results achieved by AMGWO with those of other competing methods. The outcomes are detailed in Table 8 and Table 9, respectively. While AMGWO may not have secured the top rank in certain datasets, it boasts the lowest Friedman mean value across 15 datasets and emerges as the overall top performer. This underscores the superior performance of our proposed AMGWO over other algorithms considered in the selected datasets.

**Table 8:** Friedman test for classification error rate, ranked first results are shown in bold.

Datasets	GWO	GNHGWO	BABCGWO	SOGWO	EGWO	AGWO	AMGWO
Yale	3.65	2.70	4.35	4.35	5.80	4.65	**2.50**
ORL	2.70	4.10	4.60	3.90	6.50	4.00	**2.20**
COIL20	4.15	3.25	4.90	4.00	5.50	3.60	**2.60**
Colon	**3.15**	3.20	3.85	3.75	5.25	5.55	3.25
SRBCT	4.70	3.95	3.50	**3.15**	4.30	4.45	3.95
warpAR10P	**3.00**	3.80	4.50	3.70	5.20	4.50	3.30
warpPIE10P	3.40	**3.35**	4.15	4.40	4.65	4.15	3.90
DBWorld	3.80	5.10	3.55	3.45	5.60	4.85	**1.65**
Leukemia1	4.70	2.85	4.30	3.65	5.05	4.75	**2.70**
DLBCL	3.95	**3.10**	4.25	4.40	5.05	3.90	3.35
ALLAML	3.50	**2.70**	4.65	5.05	5.05	3.65	3.40
Pixraw10P	**3.60**	4.20	4.30	3.70	3.80	4.20	4.20
orlraws10P	4.30	3.90	5.25	3.65	**3.25**	4.00	3.65
Prostate	4.30	**3.15**	4.25	3.55	4.35	4.25	4.15
Leukemia2	3.75	**3.55**	3.65	4.00	3.95	3.90	5.20
Mean	3.78	3.53	4.27	3.91	4.89	4.29	**3.33**

**Table 9:** Friedman test for feature subset size, ranking first results are shown in bold.

Datasets	GWO	GNHGWO	BABCGWO	SOGWO	EGWO	AGWO	AMGWO
Yale	2.80	5.45	4.00	4.00	6.80	3.75	**1.20**
ORL	3.40	6.40	4.60	3.90	6.60	2.10	**1.00**
COIL20	3.60	6.05	4.75	3.95	6.25	2.00	**1.40**
Colon	4.50	3.10	4.60	4.65	5.30	4.85	**1.00**
SRBCT	4.35	4.70	3.95	3.80	5.40	3.70	**2.10**
warpAR10P	4.10	4.80	4.40	3.90	5.80	3.60	**1.40**
warpPIE10P	5.30	3.70	3.80	5.00	5.00	2.90	**2.30**
DBWorld	3.40	5.80	2.80	3.10	6.50	4.80	**1.60**
Leukemia1	3.60	4.70	2.80	3.70	6.00	4.60	**2.60**
DLBCL	3.50	4.80	3.60	4.20	4.90	4.00	**3.00**
ALLAML	4.00	3.50	4.80	3.80	5.20	4.10	**2.60**
Pixraw10P	4.60	3.25	3.90	**3.20**	5.50	4.30	3.25
orlraws10P	4.10	4.40	**3.00**	4.20	4.40	3.60	4.30
Prostate	4.50	4.90	**2.40**	4.30	4.50	4.20	3.20
Leukemia2	3.90	3.90	**3.10**	4.90	5.50	3.40	3.30
Mean	3.98	4.63	3.77	4.04	5.58	3.73	**2.28**

### 5.3 Ablation experiment

In order to assess the effectiveness of our proposed strategy, APCGWO, AFDBGWO, ADVGWO, the original GWO, and AMGWO fused with the three strategies underwent testing for accuracy analysis and feature number analysis. The experimental results are detailed in Table 10 and Table 11, respectively. Table 10 indicates that ADVGWO ranks second in accuracy analysis results, with a higher frequency of bold numbers, while the fusion of the three strategies, AMGWO, secures the top position. The table's last row also shows that AMGWO has the smallest average Friedman rank, signifying superior performance. In Table 11, ADVGWO also ranks second in the analysis results for the number of features, with a higher frequency of bold numbers, while AMGWO, incorporating the three strategies, maintains its top position. The average Friedman rank of AMGWO in the last row is 2.2, indicating its superior performance compared to the other competing algorithms.

**Table 10:** Comparison of classification error rates for ablation experiments, where the top-ranked results are shown in bold.

Datasets	Metrics	GWO	APCGWO	AFDBGWO	ADVGWO	AMGWO
Yale	Best	9.6774E-02	9.6774E-02	8.3333E-02	**0.0000E + 00**	3.8462E-02
	Worst	4.5455E-01	**3.0303E-01**	5.1515E-01	3.6364E-01	3.3333E-01
	Mean	2.5257E-01	1.8418E-01	2.1501E-01	1.8353E-01	**1.4619E-01**
	Std	9.7845E-02	**7.6261E-02**	1.3480E-01	1.2767E-01	1.0504E-01
ORL	Best	4.5455E-02	**0.0000E + 00**	**0.0000E + 00**	**0.0000E + 00**	**0.0000E + 00**
	Worst	1.4103E-01	1.5190E-01	1.6456E-01	1.0256E-01	**7.6923E-02**
	Mean	8.6298E-02	8.1565E-02	8.2828E-02	6.0536E-02	**4.7065E-02**
	Std	3.3444E-02	5.1460E-02	4.9650E-02	2.9871E-02	**2.3083E-02**
COIL20	Best	**0.0000E + 00**	3.4722E-03	1.0417E-02	**0.0000E + 00**	**0.0000E + 00**
	Worst	**3.1250E-02**	6.2500E-02	4.5139E-02	3.8194E-02	**3.1250E-02**
	Mean	1.8750E-02	2.2222E-02	2.2569E-02	1.6667E-02	**1.2153E-02**
	Std	1.0506E-02	1.6303E-02	1.0513E-02	1.2658E-02	**8.8525E-03**
Colon	Best	7.6923E-02	**0.0000E + 00**	**0.0000E + 00**	**0.0000E + 00**	**0.0000E + 00**
	Worst	3.8462E-01	**2.3077E-01**	3.0769E-01	3.8462E-01	**2.3077E-01**
	Mean	1.6923E-01	1.3077E-01	1.3077E-01	**1.2308E-01**	1.5385E-01
	Std	1.0757E-01	**8.9192E-02**	1.0288E-01	1.4137E-01	9.5940E-02
SRBCT	Best	**0.0000E + 00**	**0.0000E + 00**	**0.0000E + 00**	**0.0000E + 00**	**0.0000E + 00**
	Worst	1.7647E-01	**1.1765E-01**	1.7647E-01	1.7647E-01	**1.1765E-01**
	Mean	5.2941E-02	**4.7059E-02**	7.0588E-02	5.2941E-02	**4.7059E-02**
	Std	6.4736E-02	**3.7203E-02**	6.0753E-02	5.8496E-02	**3.7203E-02**
warpAR10P	Best	1.7391E-01	**1.0000E-01**	1.5789E-01	1.5789E-01	1.5385E-01
	Worst	6.5385E-01	5.7692E-01	6.1538E-01	**4.2308E-01**	5.0000E-01
	Mean	4.3316E-01	3.6425E-01	3.8307E-01	**3.1542E-01**	3.2168E-01
	Std	1.5686E-01	1.3987E-01	1.5249E-01	**8.9186E-02**	1.1140E-01
warpPIE10P	Best	7.1429E-02	4.7619E-02	4.7619E-02	4.7619E-02	**0.0000E + 00**
	Worst	2.6190E-01	**1.4286E-01**	2.1429E-01	1.9048E-01	1.6667E-01
	Mean	1.2381E-01	1.0000E-01	1.0000E-01	**9.2857E-02**	1.0714E-01
	Std	5.1189E-02	**2.9268E-02**	5.3593E-02	3.9603E-02	5.4120E-02
DBWorld	Best	**0.0000E + 00**	**0.0000E + 00**	**0.0000E + 00**	**0.0000E + 00**	**0.0000E + 00**
	Worst	6.1538E-01	4.6154E-01	4.6154E-01	3.8462E-01	**3.0769E-01**
	Mean	1.8462E-01	1.8462E-01	1.9231E-01	7.6923E-02	**3.0769E-02**
	Std	2.1817E-01	1.8912E-01	1.5061E-01	1.2027E-01	**9.7301E-02**
Leukemial	Best	6.6667E-02	**0.0000E + 00**	**0.0000E + 00**	6.6667E-02	6.6667E-02
	Worst	4.6667E-01	4.0000E-01	4.0000E-01	4.6667E-01	**3.3333E-01**
	Mean	2.6000E-01	2.0000E-01	**1.2667E-01**	2.0667E-01	1.8000E-01
	Std	1.3860E-01	1.2571E-01	1.3128E-01	1.5219E-01	**9.9629E-02**
DLBCL	Best	**0.0000E + 00**	**0.0000E + 00**	**0.0000E + 00**	**0.0000E + 00**	**0.0000E + 00**
	Worst	2.5000E-01	3.1250E-01	3.7500E-01	3.1250E-01	**1.8750E-01**
	Mean	1.3125E-01	1.4375E-01	1.7500E-01	1.6875E-01	**6.8750E-02**
	Std	8.0418E-02	1.0227E-01	1.1335E-01	1.0643E-01	**6.8782E-02**
ALLAML	Best	6.6667E-02	6.6667E-02	6.6667E-02	6.6667E-02	**0.0000E + 00**
	Worst	**2.6667E-01**	3.3333E-01	**2.6667E-01**	3.3333E-01	4.6667E-01
	Mean	1.7333E-01	2.0000E-01	**1.6000E-01**	2.0000E-01	2.0000E-01
	Std	8.4327E-02	9.4281E-02	**5.6218E-02**	8.3148E-02	1.4402E-01
Pixraw10P	Best	**0.0000E + 00**	**0.0000E + 00**	**0.0000E + 00**	**0.0000E + 00**	**0.0000E + 00**
	Worst	2.0000E-01	1.0000E-01	1.5789E-01	1.0000E-01	**5.0000E-02**
	Mean	4.5526E-02	2.5263E-02	8.0789E-02	5.5000E-02	**2.0000E-02**
	Std	5.9920E-02	3.5571E-02	5.9779E-02	4.9721E-02	**2.5820E-02**
orlraws10P	Best	**0.0000E + 00**	**0.0000E + 00**	**0.0000E + 00**	**0.0000E + 00**	**0.0000E + 00**
	Worst	**2.0000E-01**	**2.0000E-01**	**2.0000E-01**	2.5000E-01	**2.0000E-01**
	Mean	9.6637E-02	9.1930E-02	9.5526E-02	9.3660E-02	**6.0263E-02**
	Std	7.9957E-02	6.8883E-02	7.2516E-02	8.0421E-02	**6.5789E-02**
Prostate	Best	9.5238E-02	9.5238E-02	9.5238E-02	1.4286E-01	**0.0000E + 00**
	Worst	3.3333E-01	3.3333E-01	3.3333E-01	**2.3810E-01**	3.3333E-01
	Mean	2.0476E-01	2.0000E-01	2.3333E-01	1.9048E-01	**1.0952E-01**
	Std	7.7923E-02	9.2009E-02	7.2566E-02	**3.8881E-02**	9.2691E-02
Leukemia2	Best	6.6667E-02	**0.0000E + 00**	**0.0000E + 00**	**0.0000E + 00**	**0.0000E + 00**
	Worst	**2.0000E-01**	3.3333E-01	**2.0000E-01**	**2.0000E-01**	2.6667E-01
	Mean	**1.0667E-01**	1.2667E-01	1.1333E-01	**1.0667E-01**	1.2000E-01
	Std	9.6774E-02	9.6774E-02	8.3333E-02	**0.0000E + 00**	3.8462E-02
Friedman mean rank		3.32	3.05	3.19	2.85	**2.59**

**Table 11:** Feature subset size comparison for ablation experiments, and the top ranked results are shown in bold.

Datasets	Metrics	GWO	APCGWO	AFDBGWO	ADVGWO	AMGWO
Yale	Best	228.00	186.00	241.00	106.00	**96.00**
	Worst	483.00	349.00	478.00	276.00	**270.00**
	Mean	330.50	238.60	320.10	169.20	**134.90**
	Std	89.00	**50.18**	88.02	58.32	50.28
ORL	Best	196.00	159.00	214.00	105.00	**84.00**
	Worst	314.00	310.00	280.00	188.00	**151.00**
	Mean	256.60	217.20	243.60	138.50	**114.90**
	Std	34.82	48.99	25.29	23.59	**20.01**
COIL20	Best	303.00	265.00	293.00	132.00	**114.00**
	Worst	424.00	413.00	473.00	**251.00**	275.00
	Mean	354.60	352.40	373.60	195.90	**181.20**
	Std	43.83	42.92	54.71	**38.04**	52.14
Colon	Best	735.00	667.00	672.00	342.00	**327.00**
	Worst	1023.00	**1013.00**	1014.00	1047.00	1025.00
	Mean	947.80	937.50	930.80	828.30	**811.30**
	Std	**84.22**	98.39	107.37	212.19	225.66
SRBCT	Best	969.00	759.00	995.00	739.00	**615.00**
	Worst	1164.00	1195.00	1174.00	**1157.00**	1188.00
	Mean	1091.30	987.70	1091.80	**933.60**	945.50
	Std	68.00	128.61	**53.68**	142.42	186.79
warpAR10P	Best	828.00	491.00	782.00	320.00	**283.00**
	Worst	1184.00	979.00	1185.00	**949.00**	1130.00
	Mean	1039.50	778.50	959.60	701.00	**615.20**
	Std	**102.06**	193.02	153.42	192.73	235.23
warpPIE10P	Best	1080.00	924.00	895.00	**539.00**	633.00
	Worst	1227.00	1170.00	1228.00	1182.00	**1081.00**
	Mean	1135.90	1066.90	1142.30	879.60	**783.70**
	Std	**50.04**	81.64	105.03	182.42	159.58
DBWorld	Best	625.00	477.00	538.00	402.00	**336.00**
	Worst	2367.00	2364.00	1162.00	2350.00	**1069.00**
	Mean	1317.20	1028.50	686.80	963.40	**611.30**
	Std	675.51	587.45	**186.59**	673.35	291.98
Leukemial	Best	2086.00	1944.00	2333.00	1437.00	**1059.00**
	Worst	2710.00	2695.00	2705.00	**2628.00**	2665.00
	Mean	2584.40	2482.10	2526.10	**1867.40**	2091.80
	Std	188.13	228.18	**128.95**	376.80	507.03
DLBCL	Best	1623.00	785.00	**697.00**	1387.00	1072.00
	Worst	2743.00	**2713.00**	2785.00	2764.00	2773.00
	Mean	2394.50	**2002.50**	2096.10	2251.80	2106.50
	Std	**455.75**	766.95	788.14	502.48	599.87
ALLAML	Best	1302.00	1823.00	**846.00**	1031.00	1113.00
	Worst	3581.00	3609.00	3625.00	**3574.00**	3631.00
	Mean	3144.70	3386.50	2594.30	**2462.40**	3013.20
	Std	843.87	**550.33**	1011.93	1056.49	1013.41
Pixraw10P	Best	3376.00	1758.00	3619.00	1877.00	**1068.00**
	Worst	5132.00	5151.00	5097.00	5149.00	**5059.00**
	Mean	4499.60	4260.50	4827.20	3881.40	**3145.70**
	Std	662.94	1303.87	**444.70**	1372.94	1695.92
orlraws10P	Best	3470.00	4976.00	3755.00	3073.00	**1718.00**
	Worst	**5208.00**	5215.00	5244.00	**5208.00**	5272.00
	Mean	4783.00	5133.90	4735.30	4463.70	**4418.90**
	Std	623.74	**69.59**	565.21	828.88	1314.51
Prostate	Best	1834.00	4855.00	4511.00	2997.00	**1091.00**
	Worst	5392.00	5263.00	5336.00	5334.00	**5235.00**
	Mean	4646.20	5058.70	5128.70	4654.70	**3904.00**
	Std	1087.87	**151.93**	242.52	813.25	1239.36
Leukemia2	Best	3734.00	5332.00	4111.00	4450.00	**3592.00**
	Worst	5680.00	5671.00	5697.00	**5655.00**	5683.00
	Mean	5361.30	5554.10	5327.90	5353.20	**4893.30**
	Std	588.95	**120.78**	478.39	457.96	785.77
Friedman mean rank		3.7	3.24	3.47	2.46	**2.2**

In summary, although a single improvement strategy can achieve good results in some data sets, the comprehensive performance of AMGWO fusing three strategies is better.

## 6. Summary and outlook

This study introduces an effective FS method based on the GWO algorithm for classification purposes. Through comparative analysis with the original GWO and its five advanced variants using 15 high-dimensional datasets, the experimental findings demonstrate that the proposed AMGWO offers advantages in terms of accuracy, convergence speed, and feature subset size. These advantages can be attributed to three key aspects:

(1) The incorporation of a nonlinear parameter control strategy that effectively balances exploration and exploitation.
(2) The introduction of an adaptive fitness distance balance mechanism to prevent premature convergence in the search process and select high-potential solutions, thereby enhancing search efficiency.
(3) The development of an adaptive neighborhood mutation mechanism that takes into account the information exchange between α, β, δ wolves and the current global optimal solution, enabling the algorithm to more effectively identify the global optimal solution.

While the proposed method has demonstrated its effectiveness on high-dimensional datasets, it does have some limitations. For example, sometimes the convergence speed is insufficient and it will fall into local optimum. In addition, the research on population initialization and boundary control is not deep enough. In our future work, we intend to introduce a multi-objective version based on the GWO algorithm to tackle the multi-objective FS problem. The goal is to maximize classification performance and minimize the number of selected features simultaneously. Additionally, ensuring the algorithm's adaptability and computational efficiency across different scenarios presents a significant challenge in the field of FS.
